# Lignin-Based Polymer Electrolyte Membranes for Sustainable Aqueous Dye-Sensitized Solar Cells

**DOI:** 10.1021/acssuschemeng.1c01882

**Published:** 2021-06-14

**Authors:** Juan Carlos de Haro, Elisavet Tatsi, Lucia Fagiolari, Matteo Bonomo, Claudia Barolo, Stefano Turri, Federico Bella, Gianmarco Griffini

**Affiliations:** † Department of Chemistry, Materials and Chemical Engineering “Giulio Natta”, Politecnico di Milano, Piazza Leonardo da Vinci 32, 20133 Milano, Italy; ‡ Department of Applied Science and Technology, Politecnico di Torino, Corso Duca degli Abruzzi 24, 10129 Torino, Italy; § Department of Chemistry, NIS Interdepartmental Centre and INSTM Reference Centre, Università degli Studi di Torino, Via Pietro Giuria 7, 10125 Torino, Italy; ∥ ICxT Interdepartmental Centre, Università degli Studi di Torino, Via Lungo Dora Siena 100, 10153 Turin, Italy; ⊥ National Interuniversity Consortium of Material Science and Technology (INSTM), Via Giuseppe Giusti 9, 50121 Firenze, Italy

**Keywords:** lignin, membrane, biobased, lignocellulosic biomass, dye-sensitized solar cells, aqueous solar cells, stability

## Abstract

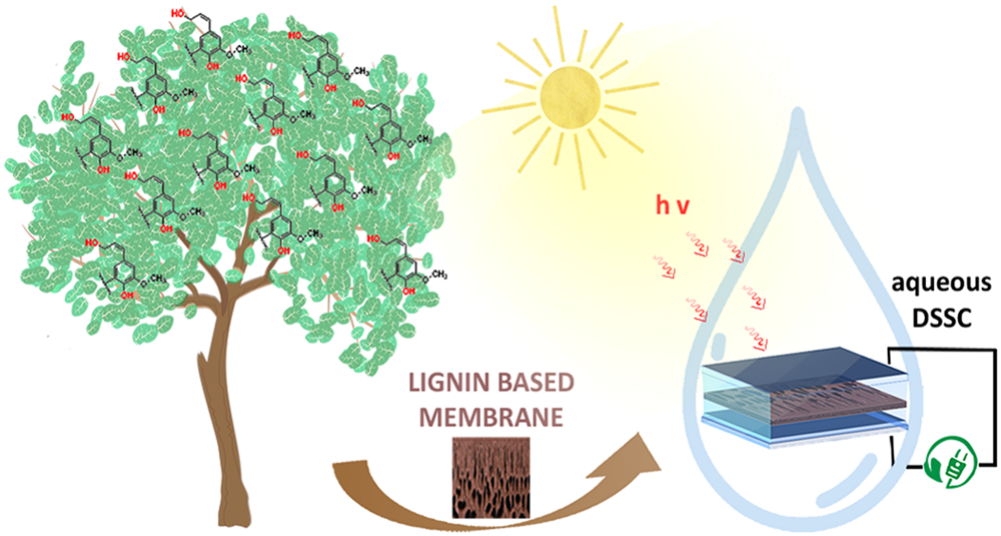

In the quest for sustainable materials for quasi-solid-state (QS) electrolytes in aqueous dye-sensitized solar cells (DSSCs), novel bioderived polymeric membranes were prepared in this work by reaction of preoxidized kraft lignin with poly(ethylene glycol)diglycidylether (PEGDGE). The effect of the PEGDGE/lignin relative proportions on the characteristics of the obtained membranes was thoroughly investigated, and clear structure–property correlations were highlighted. In particular, the glass transition temperature of the materials was found to decrease by increasing the amount of PEGDGE in the formulation, indicating that polyethylene glycol chains act as flexible segments that increase the molecular mobility of the three-dimensional polymeric network. Concurrently, their swelling ability in liquid electrolyte was found to increase with the concentration of PEGDGE, which was also shown to influence the ionic transport efficiency within the membrane. The incorporation of these lignin-based cross-linked systems as QS electrolyte frameworks in aqueous DSSCs allowed the preparation of devices with excellent long-term stability under UV–vis light, which were found to be superior to benchmark QS-DSSCs incorporating state-of-the-art carboxymethylcellulose membranes. This study provides the first demonstration of lignin-based QS electrolytes for stable aqueous DSSCs, establishing a straightforward strategy to exploit the potential of lignin as a functional polymer precursor for the field of sustainable photovoltaic devices.

## Abstract 2

Lignin-based membranes were successfully prepared and employed as quasi-solid-state electrolytes in sustainable aqueous dye-sensitized solar cells.

## Introduction

The progressive reduction of fossil resources and the severe environmental problems associated with their extensive use are pushing both academia and industry to resort to different routes for the development of more sustainable added-value products and processes. Among the number of different renewable feedstocks currently investigated for the production of biobased materials and biofuels, lignocellulosic biomass is considered one of the most promising alternatives to traditional fossil sources.^1^ Lignocellulose is principally composed by cellulose, hemicellulose, tannin, and lignin, the latter being the most abundant aromatic polymer naturally available on Earth.^2^ At industrial scale, lignin is mainly produced as a byproduct during the delignification of lignocellulose in the paper-making process. In spite of its potential as functional biopolymer, most of the lignin produced worldwide is currently burned as a low-value fuel for energy recovery,^3^ while only a little fraction of it is used as filler, additive, or dispersant in some specific formulations.^4^ Based on these considerations, one way to profitably exploit the full potential of such low-cost, naturally occurring biobased polymers is to develop new lignin-based functional materials for technological applications of increased value. Given the high amount and variety of functional groups present in the chemical structure of lignin, its direct utilization or its chemical/physical functionalization to achieve additional properties represents economically interesting and effective routes to produce biobased products with high added-value, which rely on macromolecular lignin as core building blocks.^5^ Some of the most investigated chemical pathways or structural modifications to obtain lignin-based materials and polymers include esterification,^6−8^ phenolation,^9,10^ urethanization,^11,12^ and etherification.^13,14^

In the field of solar energy conversion, dye-sensitized solar cells (DSSCs) have proven to be among the most promising, reliable, and versatile third generation photovoltaic (PV) technologies due to their potential low-cost fabrication, favorable aesthetics, and high photon-to-current conversion efficiency.^15^ A typical DSSC device is composed of a dye-sensitized nanocrystalline TiO_2_ photoanode, an electrolyte containing iodide/triiodide (I^–^/I_3_^–^) redox couples, and a counter electrode.^16^ The electrolyte is one of the most crucial components in DSSCs, since it is responsible for the inner charge carrier transport between electrodes and continuously regenerates the dye during DSSC operation. The first approaches in the field proposed the use of liquid electrolytes consisting of organic solvents and a dissolved iodide/triiodide redox couple.^17^ In spite of the high efficiency and ease of preparation of these electrolytes, the use of liquid electrolytes causes some practical problems, such as leakage and volatilization of solvent, photodegradation and desorption of dye, corrosion of the counter electrode, and ineffective sealing of the cells for long-term applications.^18^ One of the existing alternatives to solve these problems is using quasi-solid-state (QS) electrolytes, in which a thermostable polymer acts as a framework to swell and hold a liquid electrolyte. Several different polymeric matrices have been studied in QS-DSSCs, including poly(acrylic acid) modified with poly(ethylene) glycol^19^ and/or polypyrrole,^20^ poly(vinylidene fluoride-*co*-hexafluoropropylene) and, in general, many copolymers containing poly(oxyethylene) glycols to form three-dimensional (3D) interconnected structures where liquid electrolytes can be adsorbed.^21^ A couple of review articles recently appeared discussing the merits and characteristics of polymeric matrices for QS-DSSCs.^22,23^ Despite most of these materials being derived from fossil resources, some bioderived alternatives based on palm oil,^24,25^ chitosan,^26^ citric acid,^27^ carrageenan,^28^ xanthan gum,^29^ and cellulose^30^ have also been proposed in the literature. On the other hand, the use of lignin as a macro-monomer precursor in polymeric frameworks for DSSCs has not been explored to date, notwithstanding its great potential in PV and renewable energy applications.^31,32^

Within the DSSC scenario, the scientific community has recently concentrated on the partial or complete substitution of organic solvents present in liquid and QS electrolytes with water.^33,34^ This challenge is currently demanding huge efforts in the materials chemistry field, and has recently led to the development of dyes allowing proper electrode wettability,^35,36^ redox pairs stable in an aqueous environment,^37,38^ and Pt-free cathodes in response to the need of accessing cheap and less impactful devices.^39,40^ The possibility of approaching a solar cell system truly resembling an aqueous photosynthetic device also passes through the long-term stability issue, which may be addressed by designing suitable bioderived polymeric matrices for the preparation of the QS electrolyte. While some early examples in this respect have appeared in the literature in the past few years,^41−43^ more efforts are needed in the development of biobased systems as QS electrolytes for aqueous DSSCs, possibly targeting particularly aggressive stressors such as the continuous exposure to harmful UV light.

Within this framework, this study reports on the preparation and characterization of hydrophilic lignin-based membranes produced by cross-linking a preoxidized kraft lignin with poly(ethylene glycol) diglycidyl ether (PEGDGE) and on their application as QS electrolytes in aqueous DSSCs. The effect of PEGDGE/lignin relative proportions on the chemical, physical, and functional characteristics of the obtained membranes was thoroughly investigated, and clear structure–property correlations were highlighted. To provide evidence of the suitability of such lignin-based materials as sustainable QS electrolyte components in aqueous DSSCs, PV devices incorporating these systems were fabricated and evaluated in terms of both light-to-electricity conversion efficiency and lifetime under prolonged aging conditions. This represents the first demonstration of lignin-based QS electrolytes for application in stable aqueous DSSCs.

## Experimental Section

### Materials

The softwood kraft lignin used in this work (Indulin AT) was supplied by Ingevity. Poly(ethylene glycol) diglycidyl ether (PEGDGE, Mn 500 g/mol), ferrous chloride tetrahydrate (FeCl_2_·4H_2_O, analytical grade), hydrogen peroxide (H_2_O_2_, 30 wt % in H_2_O), dimethyl sulfoxide (DMSO), and sulfuric acid were provided by Sigma-Aldrich and used without any further purification. Sodium iodide (NaI), iodine (I_2_), chenodeoxycholic acid (CDCA), chloroplatinic acid (H_2_PtCl_6_), ethanol, acetone, *tert*-butanol (*t*-BuOH), sodium carboxymethylcellulose (NaCMC), and acetonitrile (ACN) were purchased from Sigma-Aldrich. Deionized water (DI-H_2_O, 18 MΩ cm^–1^ at 25 °C) was obtained by Direct-Q 3 UV Water Purification System (Millipore). 2-[{4-[4-(2,2-diphenylethenyl)phenyl]-1,2,3,3a,4,8b-hexahydrocyclopento[b]indole-7-yl}methylidene]-cyanoacetic acid (D131) was purchased from Inabata Europe S.A. Fluorine-doped tin oxide (FTO) glass plates (sheet resistance 7 Ω s*q*^–1^, purchased from Solaronix) were cut into 2 cm × 1.5 cm sheets and used as substrates for the fabrication of both the photoanodes and the counter electrodes.

### Procedures

#### Lignin Oxidation via a Fenton Pathway

The oxidation of kraft lignin was performed following the Fenton pathway by suspending a known amount of lignin in deionized water in a glass beaker (4 mL water per gram of lignin). After 30 min of stirring, FeCl_2_·4H_2_O (0.5 mmol FeCl_2_/g lignin) was added and the suspension was stirred for 30 extra minutes. Then, H_2_O_2_ was added dropwise to reach a concentration within the 0.0–1.5 vol % range, and after 24 h of continuous stirring at ambient temperature, the oxidized lignin was air-dried at room temperature.

#### Cross-Linking with PEGDGE

Cross-linking of unmodified and preoxidized lignin was performed with PEGDGE following a procedure previously proposed in the literature with some modifications.^44^ Briefly, lignin was dissolved in 3.3 M aqueous NaOH solution, and once the solution reached 50 **°**C, a certain amount of PEGDGE (0.5–2 g lignin/g of PEGDGE) was added dropwise and the system was allowed to react for 12 h. The resulting alkaline cross-linked membranes were neutralized with 0.1 N sulfuric acid, thoroughly washed with deionized water, and dried under vacuum at 50 **°**C.

#### Aqueous Solar Cell Fabrication

100% aqueous DSSCs were prepared following a procedure similar to the one described in our recent publication.^45^ D131-sensitized photoanodes and cathodes based on Pt-coated FTO glasses were used, carrying out the TiCl_4_ treatment on the semiconductor surface. For cell assembly, each lignin-based membrane was swollen for 2 h in an aqueous electrolyte (NaI 3.0 M, I_2_ 20 mM, CDCA-saturated DI-H_2_O) and then sandwiched between photoanode and cathode. The electrodes were clipped together and a cyanoacrylate glue was used as a sealant. The self-standing lignin-based membrane serves also as spacer between photoanode and cathode. The cell was pressed for few seconds with two binder clips, which were then removed upon adhesion.

## Results and Discussion

### FTIR

**Figure 1:**
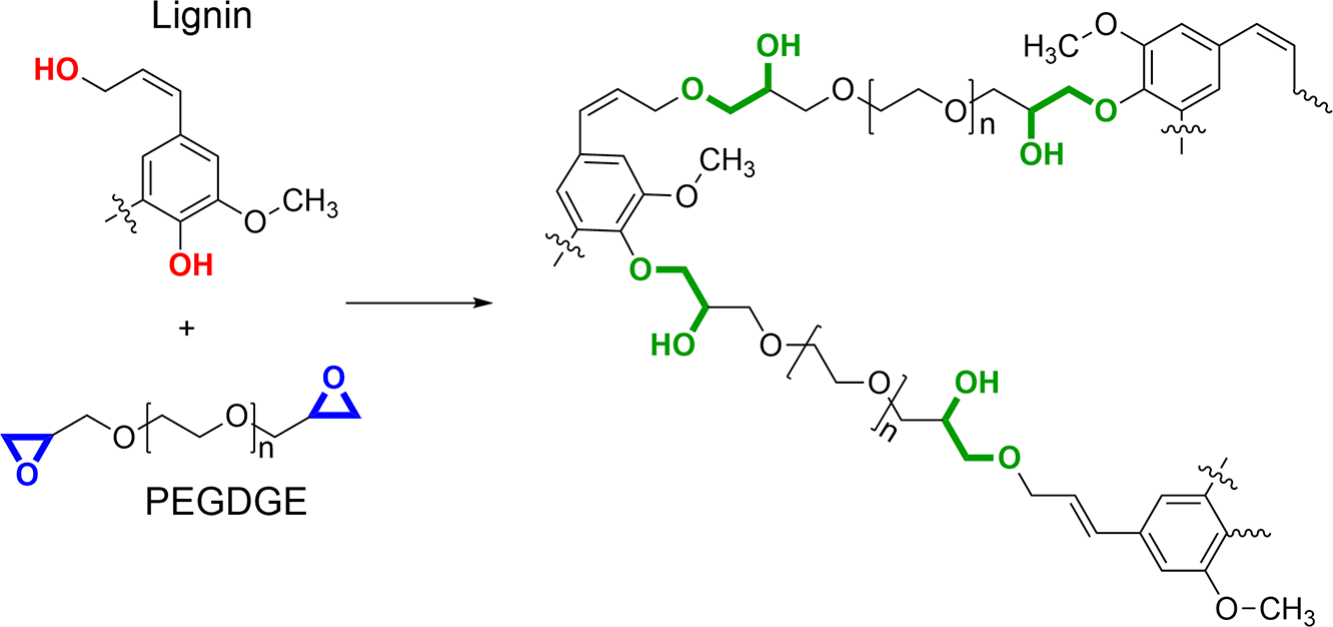
Reaction scheme of PEGDGE-mediated cross-linking of lignin under alkaline conditions.

A preoxidation step on kraft lignin (PL) via the Fenton process was performed in order to increase its reactivity toward PEGDGE, ultimately producing mechanically stable, thin hydrophilic membranes via epoxy ring-opening reaction (Figure 1).

**Figure 2:**
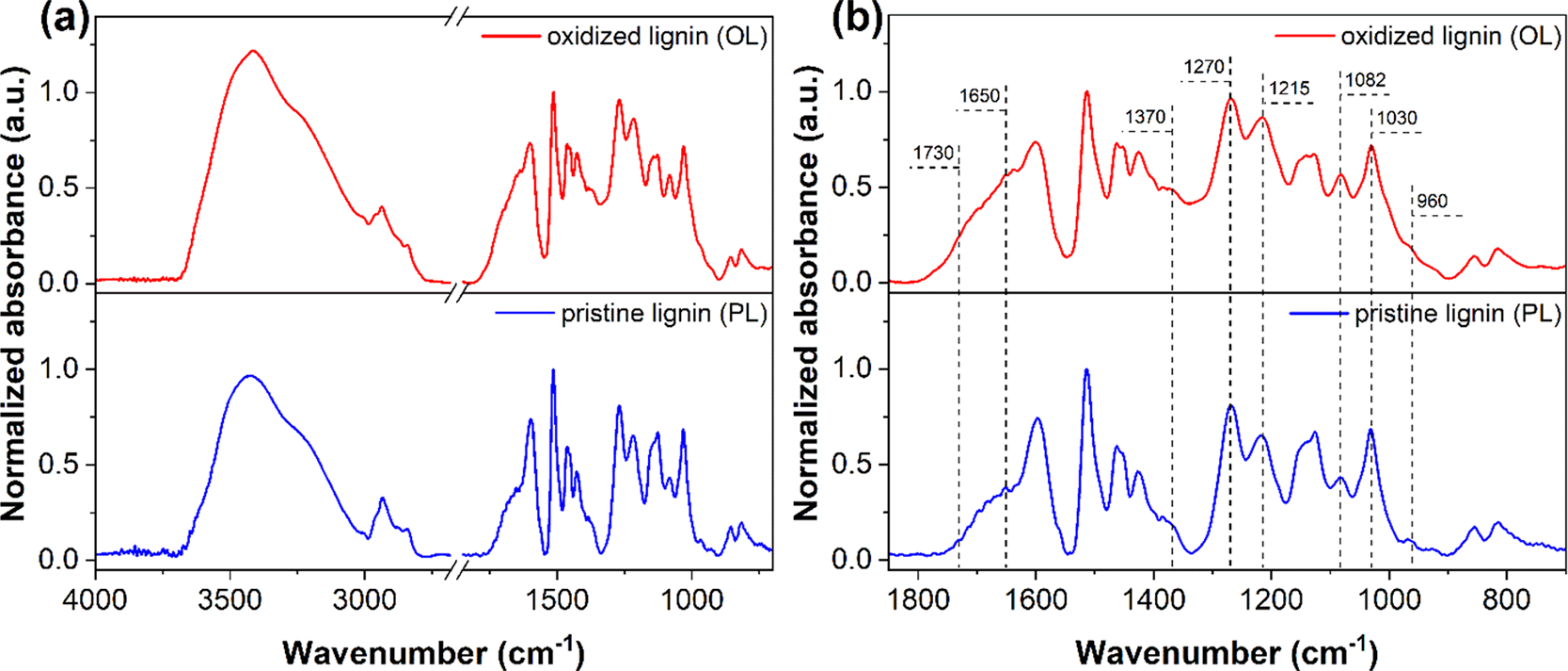
(a) FTIR comparison between PL and FL and (b) enlarged view of the fingerprint region. The spectra were normalized to the absorbance of the signal peaked at 1510 cm^–1^ (pure-aromatic skeletal vibrations in lignin), taken as an invariant band.

After the preoxidation treatment via the Fenton process, the oxidized lignin (OL) was dried and analyzed by FTIR. Figure 2 shows the FTIR spectrum of OL compared to that obtained from untreated PL. The pristine material presents a broad absorption band between 3700 and 3000 cm^–1^, corresponding to the stretching vibration of −OH moieties. This signal is found to become more intense and broader in OL. While contribution from water vapor present in the surrounding environment during measurements cannot be excluded in principle (please refer to the Experimental section for details on the FTIR analysis), such relative changes likely indicate the formation of new hydroxyl moieties upon oxidation. Other signals related to the presence of oxidated functional groups were found to be more intense in FTIR spectra for OL than for PL, namely those ascribed to stretching vibrations of aliphatic nonconjugated C=O groups at 1730 cm^–1^ (broadening of the shoulder and increased intensity), stretching vibrations of carbonyl double bonds in 4-alkyl phenol units at 1650 cm^–1^, in-plane vibrations of phenolic −OH groups at 1370 cm^–1^ (higher signal intensity), C=O and C–O stretching vibrations of guaiacyl units at 1270 and 1215 cm^–1^, respectively, and C–O deformations of secondary and primary alcohols at 1082 and 1030 cm^–1^, respectively. Additionally, the reduction of the intensity of the signal related to the aliphatic −HC=CH– out-of-plane deformations at 960 cm^–1^ may indicate partial cleavage of the double bonds in the phenyl-propane unit of lignin upon interaction with the hydroxyl radicals generated during the Fenton process, as previously observed on analogous model systems.^46,47^

### ^31^P NMR

^31^P NMR analyses were performed in order to quantitatively determine the effect of the Fenton oxidation process on the abundance of −OH moieties in PL and OL (^31^P NMR spectra are presented in Figure S1 in the Supporting Information). As reported in Table 1, unmodified PL showed values of 2.11 and 3.55 mmol/g for aliphatic and phenolic hydroxyl groups, respectively, together with 0.33 mmol/g for carboxylic acid groups. These values are in line with those found in previous literature reports on the same type of lignin.^48^ As expected, after the Fenton oxidation process, an overall increase in the concentration of these functional groups was observed, leading to a total content of hydroxylated moieties (hydroxyls and carboxyls) of 7.58 mmol/g. In particular, a notably higher concentration of phenolic hydroxyl groups was found in OL (4.56 mmol OH/g lignin) compared to PL, likely associated with a partial cleavage of β-O-4 structures in lignin during the oxidation process as a result of the interaction with the formed hydroxyl radicals.^46^ Further evidence of such cleavage is provided by the slightly reduced molecular weight and polydispersity of OL compared with PL (Table 1 for the values of Mn and PDI, Figure S2 for the chromatograms in the Supporting Information).

**Table 1:** Concentration of Hydroxyl (Aliphatic, Phenolic) and Carboxylic Groups (mmol/g_lignin_) as Obtained from Quantitative ^31^P NMR Analysis; Number Average Molecular Weight (Mn) and Polydispersity Index (*Đ)* as Obtained from GPC Analysis; Reported Values Refer to Pristine (PL) and Oxidized (OL) Lignins

	OH content (mmol/g)					
sample	aliphatic (Aliph-OH)	phenolic (Ph-OH)	carboxylic (−COOH)	total^a^	Mn (g/mol)	*Đ*
PL	2.11	3.55	0.33	5.99	1810	2.3
OL	2.59	4.56	0.43	7.58	1460	1.9

a Aliph-OH, Ph-OH, and −COOH groups were considered.

### Thermal Characterization

**Figure 3:**
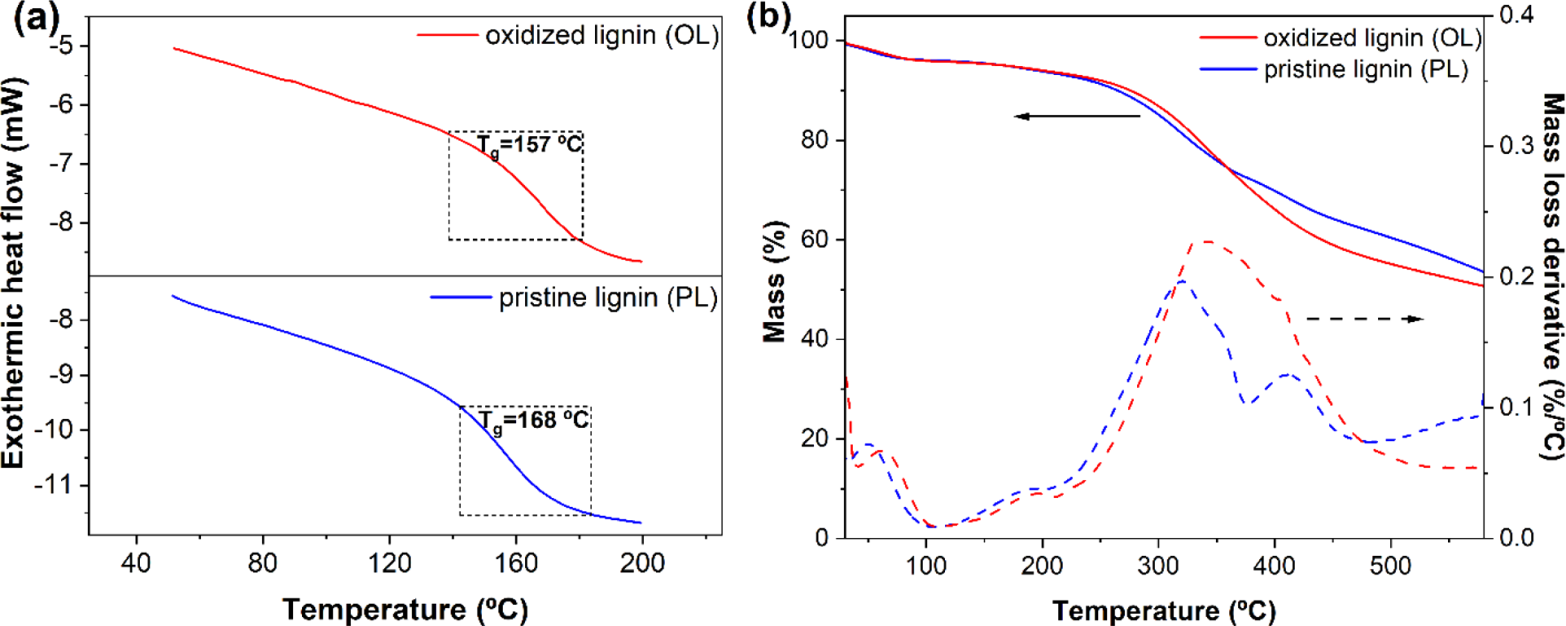
(a) DSC traces and (b) TGA profiles of PL and OL.

DSC analysis on PL and OL revealed an increase in the *T*_g_ of lignin upon oxidation from 157 to 168 °C, respectively (Figure 3a). This evidence suggests a reduction of the molecular mobility in OL with respect to PL, likely arising from increased intra- and intermolecular interactions due to the formation of additional hydrogen bonds between hydroxy and carboxy moieties generated during the Fenton process. These increased interactions appear to overcome the partial plasticization effect expected from the slightly reduced molecular weight after oxidation. To evaluate the thermolytic response of lignin before and after the Fenton process, TGA measurements under N_2_ atmosphere were performed (Figure 3b). PL exhibited a three-stage thermal degradation profile, with major mass losses in the 40–100, 150–350, and 350–500 °C temperature ranges. The first mass loss can be related to the evaporation of entrapped solvent and water, leading to a weight reduction of ∼4 wt % (also observed in OL).

A second degradation step associated with the breaking of α- and β-aryl-alkyl ether linkages and aliphatic chains^49^ led to an additional 20 wt % loss, followed by a third step (10 wt % loss) related to the complete rupture of C–C bonds in lignin. On the contrary, in the case of OL, a broader and more comprehensive mass loss event was observed in the 150–500 °C temperature range, indicating more complex and heterogeneous thermolytic degradation pathways occurring in the oxidized system. This behavior may be attributed to the higher concentration and variety of functional groups and oxidized moieties present in FL with respect to OL, broadening the range of occurrence for this thermolytic process.

### Lignin-Based Membranes

Based on the obtained Fenton-modified lignin platform, different membrane materials were obtained by the cross-linking reaction between OL and PEGDGE at increasing OL/PEGDGE mass ratios, namely 0.5, 0.7, 1, and 2 g_OL_/g_PEGDGE_ (these lignin-based membranes were named LM_0.5, LM_0.7, LM_1, and LM_2, respectively). Based on the molar amount of both hydroxyl and carboxyl −OH moieties in lignin, these mass ratios correspond to OH/epoxide equivalent ratios of 0.88, 1.17, 1.75, and 3.51 mol/mol. It is worth highlighting that with the use of OL no issues were found on the mechanical integrity of the obtained membranes, as opposed to previous findings on systems based on PL. This evidence further confirms the positive effect of the Fenton oxidation pretreatment on lignin reactivity. In particular, successful cross-linking was demonstrated through solvent resistance tests performed by submerging the obtained LM systems in DMSO (a good solvent for both lignin and PEGDGE) for 24 h, which resulted in residual gel content of over 97% for all OL-based formulations.

### FTIR

**Figure 4:**
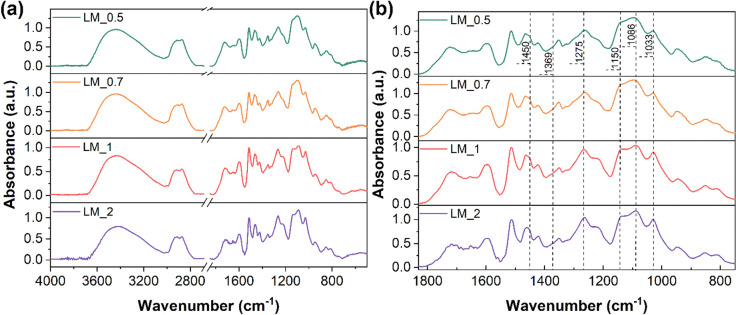
(a) FTIR spectra of lignin-based membranes and (b) enlarged view of the fingerprint region. The spectra were normalized to the absorbance of the signal peaked at 1510 cm^–1^ (pure-aromatic skeletal vibrations in lignin), taken as invariant band.

To investigate the chemical structure of the materials resulting from reaction of OL and PEGDGE in different proportions, FTIR spectroscopy was used (Figure 4). All LMs presented an absorption band between 3700 and 3000 cm^–1^ corresponding to the stretching vibration of −OH groups, whose broadness was found to reduce by increasing the amount of PEGDGE. As the spectral breadth of this signal is typically related to the presence of a wide variety of −OH moieties in the material, its reduction may indicate a decrease in the variety of such groups. This evidence is in line with the reaction mechanism previously proposed (Figure 1), in which increasing PEGDGE content during the cross-linking process may favor the consumption of primary aliphatic and phenolic hydroxyl groups from OL, leading to the generation of secondary aliphatic −OH moieties as a product of the epoxy ring-opening reaction. This mechanism can be confirmed by the disappearance of the characteristic signals associated with the oxirane ring vibrations (950–750 cm^–1^ region) in PEGDGE (FTIR spectrum in Figure S3 in the Supporting Information) and by the signal associated with the C–O deformations of secondary alcohols at 1086 cm^–1^, whose intensity is found to increase during the cross-linking process at higher PEGDGE concentrations. In addition, the bands ascribed to C–O stretching and in-plane vibrations of phenolic groups at 1275 and 1369 cm^–1^ (shoulder), respectively, and the C–O stretching vibrations of primary aliphatic hydroxyl groups at 1033 cm^–1^ presented a lower intensity in these cases. Finally, successful covalent incorporation of OL in PEGDGE was also confirmed by the increased intensity of the absorption bands at 1150 cm^–1^ (asymmetric stretching vibration of C–O–C bonds in CH_2_–O–CH_2_ units), in the 2980–2820 cm^–1^ range (stretching vibration of aliphatic methylene groups) and at 1450 cm^–1^ (deformation of C–H bonds in −CH_2_– units).

### Thermal Characterization

**Figure 5:**
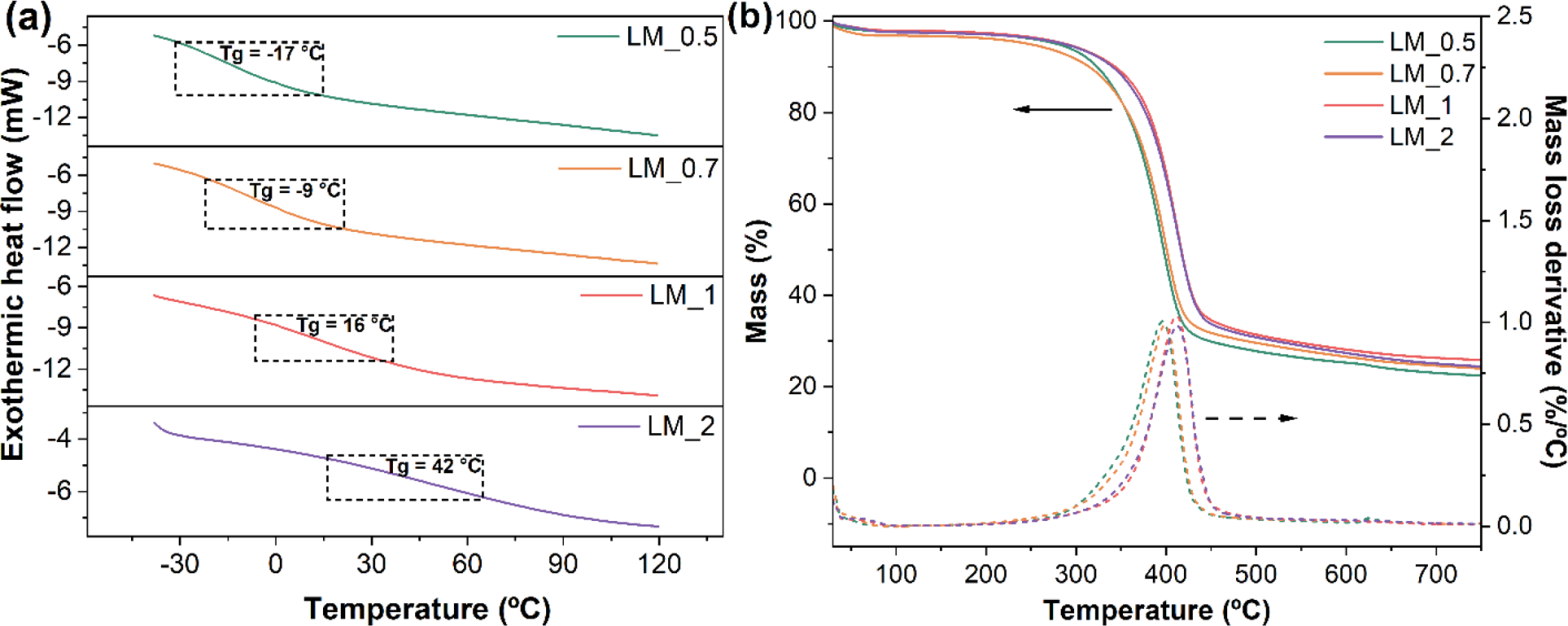
(a) DSC traces and (b) TGA profiles of lignin-based membranes.

The calorimetric characteristics of the lignin-based membranes were assessed by means of DSC (Figure 5a) analysis. All systems presented a single thermal transition, indicating that the materials are homogeneous without any indication of phase segregation. Upon reaction with PEGDGE (*T*_g_ = −67 °C, Figure S4 in the Supporting Information), a decrease of *T*_g_ compared to that of OL was observed in all systems, with values ranging from −17 to 42 °C. This reduction was more marked in membranes incorporating higher amounts of PEGDGE (i.e., LM_0.5 and LM_0.7), indicating that polyethylene glycol chains act as flexible segments that increase the molecular mobility of the 3D polymeric network, as also observed on other biobased systems incorporating ethylene glycol derivatives as cross-linkers.^50^

The thermolytic response of the membranes was investigated by means of TGA measurements in inert (i.e., N_2_) atmosphere (Figure 5b). All OL-derived materials were found to exhibit a higher thermal stability than the parent lignin, indicating a positive effect of the cross-linking process as a result of the presence of the 3D macromolecular structure.^7^ In addition, in all cases a single-step thermal decomposition was observed in the 300–500 °C temperature range, which appeared to initiate at slightly lower temperatures (∼20 °C lower) in systems incorporating higher PEGDGE concentration (LM_0.5 and LM_0.7) likely due to the negative effect of ethylene glycol moieties on the thermal stability of the material, as previously described in the literature (the TGA thermogram of PEGDGE is reported in Figure S5 in the Supporting Information).^50^

### DSSCs Characterization

**Figure 6:**
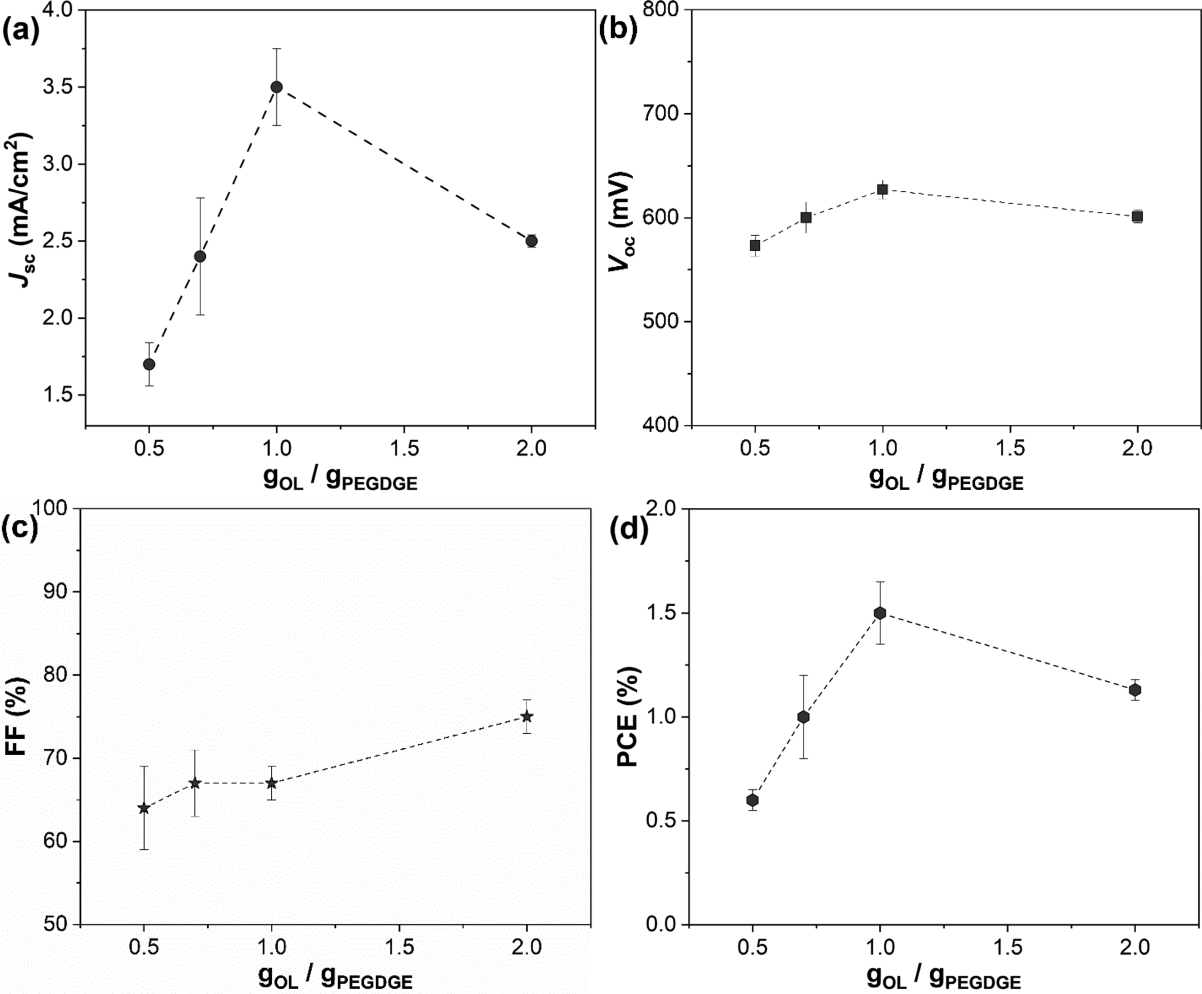
PV parameters for aqueous DSSCs assembled with lignin-based QS electrolytes at varying OL/PEGDGE weight ratio: (a) *J*_SC_; (b) *V*_OC_; (c) FF; (d) PCE. Each point represents the average of 5 devices, measured under 1 sun AM 1.5G irradiation (error bars represent the standard deviation).

The ability of the lignin-based cross-linked membranes to retain water upon soaking was evaluated in terms of their free swelling capacity (FSC), whose values were found to lie within the typical ranges of common polymeric membranes used for traditional DSSC electrolytes (Figure S6 in the Supporting Information).^51,52^ Accordingly, lignin-based QS polymer electrolytes were obtained by soaking lignin-based membranes into an aqueous solution containing the iodide/triiodide redox shuttle for 2 h, i.e., the time required to achieve the maximum liquid uptake based on the FSC tests discussed previously. The resulting wet membranes were sandwiched between DSSC electrodes and tested under an LED-powered sun simulator. Short-circuit current density (*J*_SC_), open-circuit voltage (*V*_OC_), fill factor (FF), and power conversion efficiency (PCE) were extracted from *J–V* curves and their average values (for batches consisting of five devices each) are shown in Figure 6, as a function of the OL/PEGDGE weight ratio. Clearly, *J*_SC_ was found to raise sharply by increasing the OL/PEGDGE weight ratio, reaching a maximum value at OL/PEGDGE = 1 wt/wt Conversely, *V*_oc_ and FF values were found to remain rather constant by varying PEGDGE content. The best device showed a PCE = 1.54%, resulting from *J*_SC_ = 3.62 mA/cm^2^, *V*_OC_ = 634 mV, and FF = 0.67 (for OL/PEGDGE = 1 wt/wt). Interestingly, a remarkably high FF ≈ 0.75 was measured for OL/PEGDGE = 2.0 wt/wt, indicative of the rather good assembly of our lab-scale DSSCs. Based on these trends, solar cell efficiency was found to be primarily determined by the pronounced dependence of *J*_sc_ on OL/PEGDGE relative proportions (Table 2). To make a comparison with liquid-state devices, the corresponding liquid-state counterpart was also fabricated and tested (*J*_SC_ = 4.40 mA/cm^2^, VOC = 610 mV, FF = 0.58, and PCE = 1.50%).^53^ Moreover, the incident photon-to-current efficiency (IPCE) spectra of both liquid-state and the most efficient QS-DSSC (i.e., LM_1) were compared and are shown in Figure S7 in the Supporting Information. *J*_SC_ values calculated from the overlap integral of the IPCE spectra with the standard AM 1.5G solar emission spectrum were 3.69 and 4.38 mA/cm^2^ for cells assembled with QS and liquid-state electrolytes, respectively, in good agreement with values obtained from *J*–*V* experiments. The different trends observed in the IPCE traces can be associated with both the UV-absorbing feature (left side of the spectrum) and the cross-linked nature of the lignin-based QS electrolyte, as will be thoroughly discussed in the following paragraphs. It is worth highlighting that device performance could be further improved with a more sophisticated cell design, as we recently demonstrated by tailoring the TiO_2_ electrode composition^53^ and by replacing iodine with cobalt as the redox mediator,^54^ reaching PCE values close to 5%. However, such a device optimization strategy is out of the scope of the present investigation.

**Table 2:** PCE and *R*_d_ (Measured by Means of EIS) Values of Complete DSSC Devices, Ionic Conductivity (σ) Values of the Polymer Electrolyte Membranes Measured at 25 °C, and *X* Parameter for All Lignin-Based Membranes^a^

	PCE [%]	*R*_d_ [Ω]	σ [10^–4^ S cm^–1^]	*Χ* [S·10^–3^]
LM_0.5	0.60 ± 0.05	36.7	1.05	1.71
LM_0.7	1.00 ± 0.20	20.7	1.24	2.90
LM_1	1.50 ± 0.15	27.4	1.71	3.71
LM_2	1.13 ± 0.05	41.4	1.59	1.86

a ***X=*ṽ·φ*_PEGDGE_/ER_d_* where *ṽ* is the volume fraction of the solid membrane in the swollen material, φ_PEGDGE_ is the volume fraction of PEGDGE in the membrane, ER_d_ = *R*_d_·FSC_vol_ is the effective ionic diffusion resistance.

In the QS-DSSCs field, the nature of the cross-linked polymeric membrane is a dominating factor that heavily impacts on the performance of the resulting device assembly for a couple of important reasons. First, the extent of cross-linking of the membrane determines its mechanical properties and the handleability of the electrolyte. In turn, this influences the ease of device assembly as well as the long-term stability of the solar cell (i.e., a fully cross-linked membrane is usually able to effectively trap the liquid electrolyte for a longer time).^55^ Second, the three-dimensional macromolecular network of the membrane material should allow for a relevant electrolyte uptake while ensuring a rapid and nonlimited mass transport of the redox shuttle. This means that an excessively entangled macromolecular structure (both physically and chemically) is expected to lower the ionic conductivity of the electrolyte, thus negatively impacting *J*_SC_ values.

To gain further insights into the ionic transport characteristics of the QS electrolyte, we exploited electrochemical impedance spectroscopy (EIS) as a powerful tool to investigate mass diffusion and charge transfer processes. Typical Nyquist’s plots (i.e., real vs imaginary component of impedance *Z*) show three semicircles; the lower frequency one is associated with the ionic diffusion throughout the electrolyte. Usually, in QS-DSSCs this semicircle is rather faint^56^ due to the slackening of the diffusion kinetics in QS electrolytes. Thus, applied frequencies lower than 1 mHz should be scanned in order to obtain a complete semicircle and a clearer description of the redox shuttle transport phenomenon. As shown in Table 2, the ionic diffusion resistance (*R*_d_) of the lignin-based membranes was found to reach a minimum value for LM_0.7 (i.e., 20.7 Ω), while no clear correlations between *R*_d_ and the chemical composition of the membranes or device PCE could be highlighted, likely indicating that the PV performance of these QS-DSSC systems is in fact affected by a more complex interplay of different factors associated with the QS electrolyte system. Indeed, in addition to the ionic diffusion capability of the membrane, the spatial arrangement of the macromolecules within the 3D cross-linked structure and its hydrophilicity are also expected to play a significant role in terms of potential mass transport limitations to the redox couple. Therefore, a quantitative figure to describe this combined effect as a function of OL/PEGDGE relative proportions was sought.

Accordingly, swelling tests were performed on the different lignin-based membranes. After immersion in the aqueous electrolyte at 25 °C for 7 days (time required to achieve swelling equilibrium), the volumetric fraction of the solid membrane in the swollen material (*ṽ*) was evaluated, as an experimental measure of the ability of the formulation to entrap the liquid electrolyte (lower values of *ṽ* indicate good swellability of the membrane; see the Supporting Information for experimental details and calculations). To consider the influence of PEGDGE content on the affinity between membrane and liquid electrolyte, the volume fraction of PEGDGE (*φ*_PEGDGE_) for each membrane formulation was also calculated and introduced as correction factor for *ṽ*. The resulting term *ṽ·φ*_PEGDGE_ represents a quantitative descriptor of the volumetric contribution of PEGDGE to the swelling ability of the membrane. In addition, being an indication of the fraction of PEGDGE per unit volume of swelled membrane, *ṽ·φ*_PEGDGE_ can also be qualitatively correlated with the 3D macromolecular structure of the membrane as a function of its chemical composition. In particular, in systems exhibiting a higher lignin content (lower *ṽ·φ*_PEGDGE_), a 3D cross-linked network of (many) rigid lignin macromolecules intercalated by (few) long flexible PEG chains is formed upon direct reaction between epoxy groups in PEGDGE and hydroxyl moieties in lignin, yielding a relatively high-*T*_g_ architecture with limited affinity for the aqueous electrolyte solution (high lignin content, limited swellability). By increasing PEGDGE content (lower lignin/PEGDGE weight ratios, higher *ṽ·φ*_PEGDGE_), reaction of epoxy moieties also with secondary hydroxyls formed during the primary epoxy ring-opening process may be possible (Figure S8 in the Supporting Information), as evidenced by FTIR analysis (Figure 4). As a result, a more entangled 3D macromolecular structure may be expected, further promoted by the enhanced flexibility of the hydrophilic PEG chains. Based on these considerations, the parameter *ṽ·φ*_PEGDGE_ can be considered as a qualitative measure of how entangled the system is per unit volume. Furthermore, given that the diffusive transport of the ionic couple through the membrane is expected to be influenced by how entangled the macromolecular structure is, *ṽ·φ*_PEGDGE_ can also provide a semiquantitative description of the contribution to mass (redox shuttle) transport limitations resulting from the chemical composition and spatial arrangement of the macromolecules within the membrane.

The other major contribution to ionic transport in the system is associated with the actual amount of aqueous liquid electrolyte that can be effectively entrapped per unit volume by the membrane, which is in turn correlated with its the chemical composition of the membrane. In particular, in the absence of mass transport limitations purely linked to an excessively entangled macromolecular structure, higher ionic conductivities (lower values of *R*_d_) are to be expected in more swellable membranes. The relationship between liquid electrolyte uptake and ionic conductivity at varying OL/PEGDGE weight ratios can be rationalized in terms of an effective ionic diffusion resistance (ER_d_), expressed as the product between *R*_d_ and volumetric FSC (FSC_vol_), the latter term being introduced as a correction factor incorporating the contribution of membrane swelling ability to ionic resistance. For higher ER_d_ = *R*_d_·FSC_vol_ values, membranes with similar FSC_vol_ will exhibit higher ionic diffusion resistance values (viz., lower ionic conductivities).

Based on the characteristic figures introduced so far, the ratio between *ṽ·φ*_PEGDGE_ and ER_d_ (*ṽ·φ*_PEGDGE_/ER_d_, indicated with the letter *Χ* in the following) can be considered as a qualitative measure of the combined effect on device efficiency of macromolecular 3D structure of the membrane and ionic mass transport through the electrolyte, at varying lignin/PEGDGE relative proportions. Table 2 displays the values of *Χ* (units: S) for the different membrane formulations. Clearly, at low concentrations of lignin, the membranes possess enhanced aqueous liquid electrolyte uptake (Figure S6, Supporting Information). However, the highly entangled macromolecular structure in these high PEGDGE-content membranes leads to clear limitations in terms of mass transport of the redox couple, due to the high volumetric hindrance to ionic diffusion. Accordingly, low *Χ* values are observed. By progressively increasing the amount of lignin in the formulation, the 3D structure of the membrane becomes gradually less entangled due to the lower amount of PEGDGE present, making the transport of the redox couple increasingly less difficult and leading to higher ionic conductivities. Concurrently, the membrane becomes more rigid (its *T*_g_ increases due to the higher lignin content, Figure 5). As a result, *Χ* tends to increase. By further increasing the amount of lignin beyond a threshold value (in the present case, for lignin/PEGDGE > 1 wt/wt), the poor hydrophilicity of the membrane associated with the high lignin content is found to significantly limit the amount of aqueous liquid electrolyte uptake (lower FSC_vol_), thus yielding reduced ionic conductivity. In these conditions, the negative contribution related to the ionic diffusion resistance becomes predominant and *Χ* decreases. These trends perfectly correlate with those recorded for device PCE and for ionic conductivity values of lignin-based membranes (Table 2), further confirming the combined synergistic effect of ionic mass transport diffusion and macromolecular structure and chemistry on device performance. Interestingly, EIS analyses also reveal that the limitation of mass transport throughout the electrolyte and the 3D architecture of the membrane are the only parameters impacting on solar cell efficiency. Indeed, other key figures such as the electron transport resistance *R*_t_, the recombination resistance *R*_rec_, and the charge transfer resistance at the counter electrode *R*_CE_ remain practically constant by varying membrane formulation (Table S2, Supporting Information). Similar conclusions were drawn based on transient photocurrent measurements under different light intensities and open-circuit voltage decay (OCVD) measurements (Figure S9, Supporting Information).

**Figure 7:**
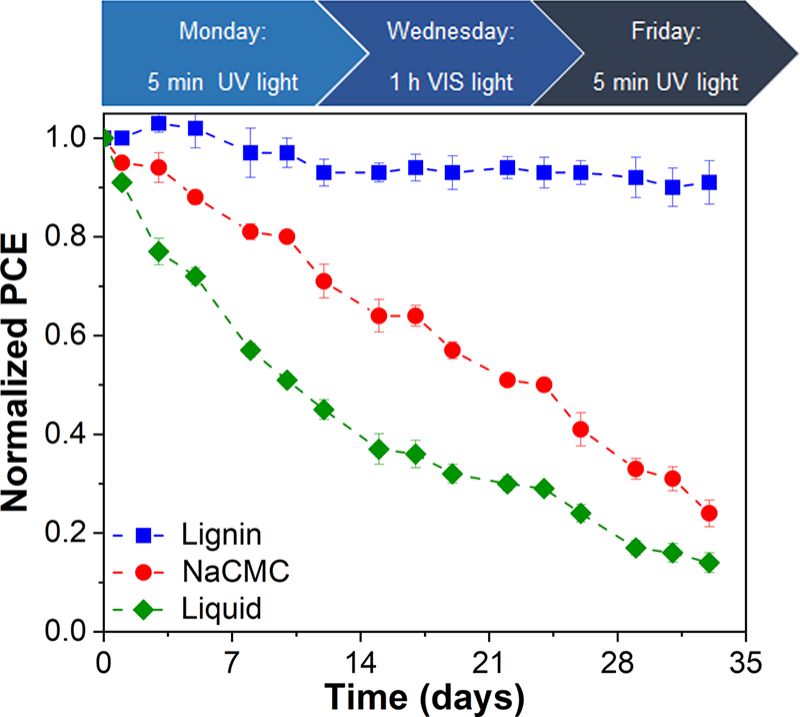
Long-term stability test for lignin-based, NaCMC-based, and liquid-state DSSCs. The aging protocol is shown in the upper part of the figure, and light irradiation was alternately directed on the photoanode- and on the cathode-side.

From a practical perspective, the long-term stability of DSSCs is knowingly improved by adopting a QS matrix as electrolyte, since leakage of the liquid electrolyte from the device assembly is largely avoided. However, a second factor to be considered when evaluating the aging of hybrid solar cells is their photochemical stability, since organic moieties present in the device components are known to undergo light-induced degradation when exposed to particular photon wavelengths or intensities. Lignin-based materials are characterized by a strong visible and UV-light absorbance.^57^ On the one hand, this feature could represent a disadvantage, since the QS electrolyte would become a competitor of the molecular sensitizer for the light harvesting process (especially for the diffuse component of incident light), thus leading to reduced photon-to-electron conversion efficiency. On the other hand, however, a lignin-based electrolyte can boost the photostability of the photoanode during prolonged irradiation, by capturing harmful wavelengths (especially in the UV range) and acting as a natural radical scavenger.^58^ To better investigate this aspect, we setup an aging test where DSSCs incorporating the QS electrolyte were systematically subjected to a combination of UV light (5 min/day, 40 mW/cm^2^) and visible light (1 h/day, 100 mW/cm^2^), following the protocol shown in Figure 7. The aging test was carried out for 5 weeks on three batches of three devices each: one set fabricated with LM_1 membrane (the one providing the highest device efficiency as reported in Figure 6), one set incorporating a QS electrolyte based on a different polymeric matrix (NaCMC), and one set based on reference liquid-state DSSCs. NaCMC was proposed by our group for water-based some years ago,^41^ demonstrating its good operation and remarkable stability and thus representing a valuable choice to gauge the properties of the lignin-based systems presented in this work. As shown in Figure 7, where the normalized PCE of lignin- and NaCMC-based DSSC devices over aging time is reported, strikingly different responses are observed for the two systems. In particular, solar cells incorporating the NaCMC electrolyte exhibit an evident efficiency drop throughout the course of the test, with peak decline during UV light exposure. This behavior can be explained considering that NaCMC cannot absorb such high-energy photons due its complete transparency in the UV-light range,^59^ thus excluding any possible UV-protective action toward the molecular dye. It is worth highlighting that NaCMC is known to be photostable under UV-light, as confirmed by its wide use as matrix system in several fields operating under UV irradiation.^60,61^ Accordingly, the observed PCE worsening upon irradiation is not to be attributed to a photodegradative effect originating from the polymeric electrolyte matrix, but more likely to the light-induced degradation of the molecular sensitizer, which is known to undergo a radical-mediated photochemical oxidation when exposed to UV light.^62^ Strikingly, liquid-state devices showed even worse long-term stability performance, due to the absence of a polymer matrix avoiding electrolyte evaporation or leakage from sealing imperfections. Conversely, the lignin-based DSSC devices exhibit extended durability under these conditions, with only a small PCE decrease (<5%) detected after 5 weeks of exposure. These results clearly demonstrate the excellent stabilizing effect of the lignin-based material system, which can be associated with a synergistic combination of different effects. On the one side, lignin is known to be a natural radical scavenger.^58^ Therefore, it can effectively quench radical species potentially forming both at the electrode/electrolyte interface and within the liquid portion of the electrolyte when the device is exposed to UV light, thereby reducing the probability of radical species to interact with the organic sensitizer and yield decreased device performance. On the other side, lignin is a strong UV-light absorber (see Figure S10, Supporting Information). This feature provides an effective protection for the sensitizer molecules in the proximity of the electrode/electrolyte interface, where there is percolation between electrolyte and adsorbed dye. Accordingly, in this region interaction between harmful high-energy photons and sensitizer molecules is in good part prevented, ultimately ensuring extended solar cell lifetime. Finally, given the cross-linked nature of the proposed lignin-based membrane, a lower molecular mobility is expected than in the case of NaCMC-based systems. As a result, the potential motion of harmful, photogenerated radical species is largely restricted with consequent limitation in their interaction with the photoactive components of the device assembly.

## Conclusions

In this work, novel bioderived polymeric membranes based on the cross-linking reaction between preoxidized kraft lignin and PEGDGE were fabricated, characterized, and employed as QS electrolytes in aqueous DSSC devices. By tuning the relative proportions of lignin and PEGDGE for the cross-linking process, fine control over the chemical and physical characteristics of the obtained membranes could be achieved. In particular, their glass transition temperature and their swelling ability were found to be strictly dependent on the amount of flexible hydrophilic PEGDGE present in the formulation, which was also demonstrated to influence the ionic transport efficiency within the membrane. The incorporation of such lignin-based cross-linked materials as QS electrolyte systems in aqueous DSSCs allowed to achieve solar cell devices with excellent long-term photostability under UV–vis light, which was found to be superior to benchmark QS-DSSCs incorporating state-of-the-art carboxymethylcellulose membranes. This behavior was attributed to the UV-protective action of lignin, which enables high-energy photons to be absorbed prior to reaching the photosensitive molecular dye within the DSSC assembly, thus preventing its photoinduced degradation and ultimately increasing the lifetime of the device under operation.

This work provides the first demonstration of lignin-based membranes as QS electrolytes in aqueous DSSCs, thus opening the path to the development of a new class of biobased polymeric structures for the field of sustainable PV devices.

## Supporting Information Available

Characterization techniques, ^31^P NMR spectra, GPC analyses, swelling tests in liquid electrolyte, cross-linking reaction scheme, EIS data, UV–vis absorption, FTIR, DSC, TGA analyses of PEGDGE, free swelling capacity, transient photocurrent measurements, OCVD curves (PDF)
