# Supplementary material for: Lignin-Based Polymer Electrolyte Membranes for Sustainable Aqueous Dye-Sensitized Solar Cells
Source: ACS Sustain Chem Eng. 2021 Jun 14;9(25):8550–60. doi: 10.1021/acssuschemeng.1c01882 (PMC8243320; doi:10.1021/acssuschemeng.1c01882)
Supplement: Supplementary file 1 — sc1c01882_si_001.pdf [file sc1c01882_si_001.pdf]

Supporting Information

Lignin-based polymer electrolyte membranes for sustainable aqueous dye-sensitized solar cells

Juan Carlos de Haro[a], Elisavet Tatsi[a], Lucia Fagiolari[b], Matteo Bonomo[c], Claudia Barolo[c,d], Stefano Turri[a,e], Federico Bella[b,e]* and Gianmarco Griffini[a,e]*

[a] *Department of Chemistry, Materials and Chemical Engineering "Giulio Natta", Politecnico di Milano, Piazza Leonardo da Vinci 32, 20133 Milano, Italy.*

[b] *Department of Applied Science and Technology, Politecnico di Torino, Corso Duca degli Abruzzi 24, 10129 – Torino, Italy.*

[c] *Department of Chemistry, NIS Interdepartmental Centre and INSTM Reference Centre, Università degli Studi di Torino, Via Pietro Giuria 7, 10125 – Torino, Italy.*

[d] *ICxT Interdepartmental Centre, Università degli Studi di Torino, Via Lungo Dora Siena 100, 10153 – Turin, Italy.*

[e] *National Interuniversity Consortium of Material Science and Technology (INSTM), Via Giuseppe Giusti 9, 50121 – Firenze, Italy.*

Corresponding Authors:

gianmarco.griffini@polimi.it; federico.bella@polito.it.

Number of pages: 19

Number of figures: 10

Number of tables: 2

S1. Characterization techniques

Fourier-transform infrared spectroscopy

Fourier-transform infrared (FTIR) spectroscopy was performed on a Nicolet 760 FTIR spectrophotometer. FTIR spectra were recorded in transmission mode at room temperature in air by recording 32 accumulated scans at a resolution of 2 cm^{-1} in the 4000−400 cm^{-1} wavenumber range. The obtained signals were normalized to the absorbance observed at 1510 cm^{-1}, which corresponds to the pure-aromatic skeletal vibrations in lignin.

Differential scanning calorimetry

Differential scanning calorimetry (DSC) was used to investigate the thermal transitions in lignins and lignin-based membranes. The measurements were performed on solid state samples (~10 mg) by using a Mettler Toledo DSC/823e instrument performing three runs (heating/cooling/heating) from -50 °C to 200 °C at a scan rate of 20 °C/min under N_2 atmosphere. The determination of the glass transition temperature (T_g) of the tested materials was based on the evaluation of the inflection point of the DSC trace in the last heating ramp.

Thermogravimetric analysis

Thermogravimetric analyses (TGA) were performed on the parent lignins as well as on the lignin-based membranes by means of a Q500 TGA system (TA instruments) from room temperature up to 700 °C at a rate of 10 °C/min. The response under both N_2 and air atmospheres was tested.

^{31}P-NMR spectroscopy

NMR experiments were carried out on a Bruker Avance 400 spectrometer. Acquisition and data treatment were performed with Bruker TopSpin 3.2 software. In order to quantify the –OH groups in the pristine lignins, ^{31}P NMR spectra were recorded by inverse gated proton decoupling

sequences using 90° pulse flip angle, with a 380-ppm spectral width, 256 scans and a relaxation delay of 5 s. Before analyses, the lignins were dried under vacuum overnight at 40 °C and derivatized according to a procedure previously reported in literature.[S1] As phosphitylation reagent, internal standard (IS) and relaxation reagent 2-chloro-4,4,5,5-tetramethyl-1,3,2-dioxaphospholane, N-hydroxy-5-norbornene-2,3-dicarboxylic acid imide and chromium (III) acetylacetonate were selected, respectively. The integration regions considered in this work were 152.8−152.5 ppm for the IS, 150.0−145.0 ppm for aliphatic hydroxyls, 145.0−137.0 ppm for phenolic hydroxyls and 137.0−134.5 ppm for carboxylic hydroxyls.

Gel permeation chromatography

Gel permeation chromatography (GPC) was used to determine the molecular weight of parent lignins. A Waters 510 HPLC chromatograph was used equipped with a Waters 2410 refractive index detector using THF as eluent. The sample to analyze (concentration in THF 2 mg/mL, dissolution volume 200 µL) was injected into a system of columns (Ultrastyragel models HR4, HR 3 and HR 2, provided by Waters) connected in series. The chromatographic analysis was performed at 30 °C and at a flow rate of 0.5 mL/min. Polystyrene standards in the 10^2-10^4 g/mol molecular weight range were used to calibrate the GPC system.

Free swelling capacity

The gravimetric free swelling capacity (FSC) of the materials was determined by placing approximately 0.3 g of dry lignin-based membrane in de-ionized water. After a certain amount of time, the swollen membrane was allowed to drip off for few minutes and the amount of absorbed water was determined gravimetrically following Equation 1, where m_{dry} and $m_{swollen}$ refer to the mass of dry and swollen membrane, respectively.

$$FSC = \frac{m_{swollen} - m_{dry}}{m_{dry}} \quad \text{(S1)}$$

Electrochemical and photoelectrochemical characterization

Solar cells performance was evaluated recording tree consecutive photocurrent density photovoltage curves on a Keithley 2420 Source Measure Unit, keeping a scan rate equal to 20 mV s^{-1}. Cells were irradiated under simulated 1 sun light intensity (100 mW cm^{-2}, AM 1.5G) after calibration by a silicon photodiode. A VeraSol-2 LED Class AAA solar simulator was used in this work.

Transient photocurrent measurements were carried out employing a modulation (on/off) of the incident light on DSSC devices, while varying the irradiation intensity from 0.2 to 1.0 sun step-by-step.

Open circuit voltage decay (OCVD) measurements were performed using a CH Instruments Inc. Model 680 potentiostat; cells were kept under constant illumination at the open-circuit conditions for 10 s, and then the illumination was interrupted and the voltage decay recorded as a function of time.

Electrochemical impedance spectroscopy (EIS) data were obtained with the same potentiostat, exploring the frequency range between 100 kHz and 0.1 Hz. The amplitude of the AC signal was 10 mV. Spectra were recorded under 1 sun illumination at applied DC potentials equal to the inverse of previously measured V_{oc} values.[S2] Z-view software was used to interpolate experimental spectra by using the transmission line (TL) model as equivalent circuit.

The ionic conductivity of lignin-based QS polymer electrolytes, sandwiched between stainless steel blocking electrodes, was measured by means of EIS under scanning frequencies ranging from 10 Hz to 1 MHz. The bulk resistance, R_b (Ω), of the samples was identified with Z-View software,

and the ionic conductivity was calculated by means of the equation $\sigma = l / (R_b \cdot A)$, where l (cm) is the film thickness and A (cm^2) the effective contact area between electrolyte and electrode.

Aging tests were carried out following a protocol described in the *Results and discussion* section, where visible and ultraviolet wavelengths were used to study the photostability of lab-scale cells. For aging test under visible light the solar simulator was used, while UV irradiation was produced by a Lightingcure spotlight source LC8 (model L9566, from Hamamatsu, wavelength range 240-400 nm, 20 mW cm^{-2}).

S2. Pre-oxidation of lignin *via* Fenton pathway

In a first exploratory step, the use of pristine kraft lignin (PL) was investigated in the attempt to produce hydrophilic membranes *via* epoxy ring-opening reaction with PEGDGE (**Figure 1** in the main text), the latter being selected as highly hydrophilic flexible comonomer, expected to provide excellent water swelling capacity to the resulting lignin-based material. However, obtaining self-standing thin films with a suitable thickness for use as membrane in DSSCs (≈15-20 µm) was not possible in this case, given the limited accessibility and reactivity of hydroxyl groups in PL,[S3,S4] which resulted in poor mechanical integrity of the obtained membranes. In order to overcome this issue and to increase the reactivity of lignin towards PEGDGE to obtain a thin membrane, a pre-oxidation process for lignin was thus undertaken. To this aim, the Fenton pathway was selected because of its process versatility (it can be carried out at room-to-moderate temperature and atmospheric pressure) and given that the required reagents are readily available, easy to store and handle, safe and environmentally friendly.[S5,S6]

In addition, this strategy has already been successfully demonstrated on different types of lignins to tune their oxidation degree[S7] or to enhance their reactivity, leading to lignin-based crosslinked systems of increased intra- and intermolecular interactions and improved mechanical and thermal response.[S8] In this context, a wide range of oxidation conditions have been proposed in the literature to obtain highly hydrophilic membranes able to retain up to 45 g H_2O/g polymer for application as soil remediation scaffolds.[S9] However, membranes with a more moderate swelling capacity are recommended in the production of DSSCs since changes on their volume during the swelling process might affect the final performance of the device, and mechanical issues in cell sealing may arise if the membrane is too thick. Hence, soft oxidation conditions (see *Experimental section* for more details) were selected and their effect on the chemical and physical characteristics of the so-obtained lignin were thoroughly investigated.

^{31}P NMR spectra

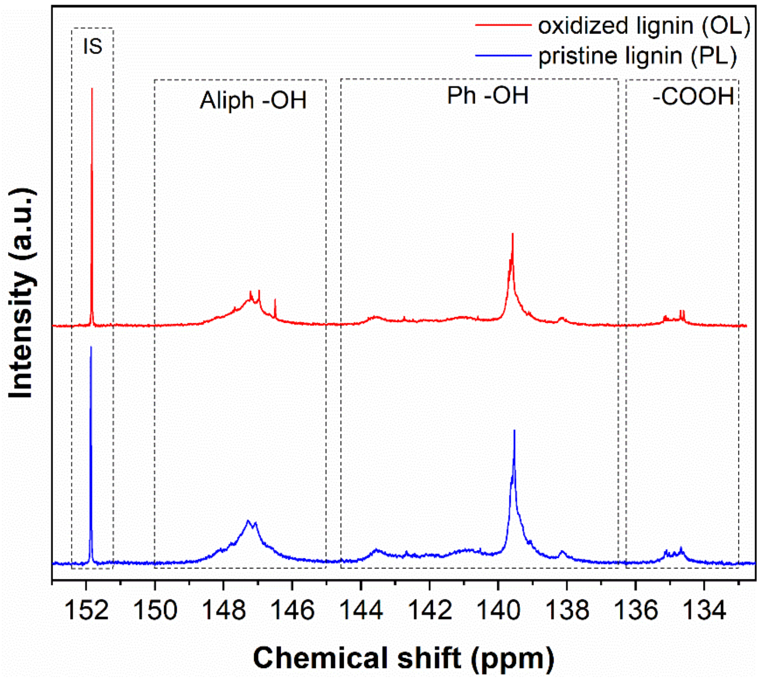

Figure S1. ^{31}P NMR spectra comparison between PL and FL**.**

GPC analyses

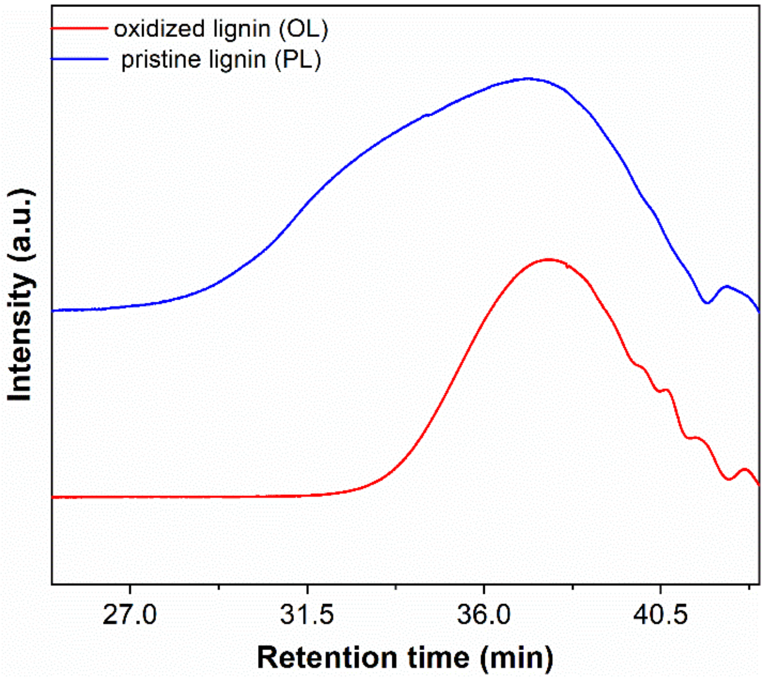

Figure S2. GPC chromatograms of parent (PL) and oxidized (OL) lignin.

FTIR analysis

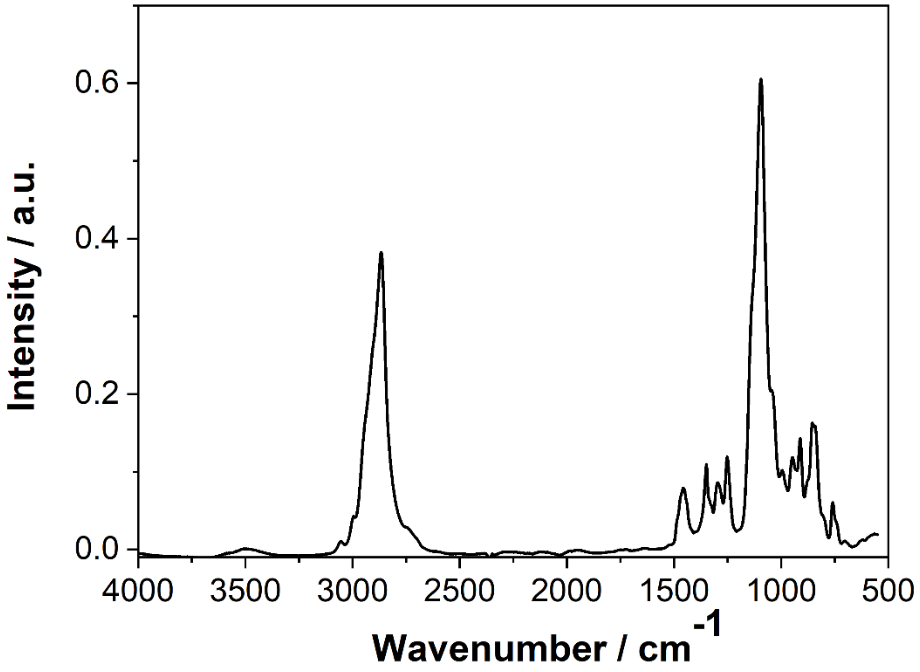

Figure S3. FTIR spectrum of PEGDGE.

DSC analysis

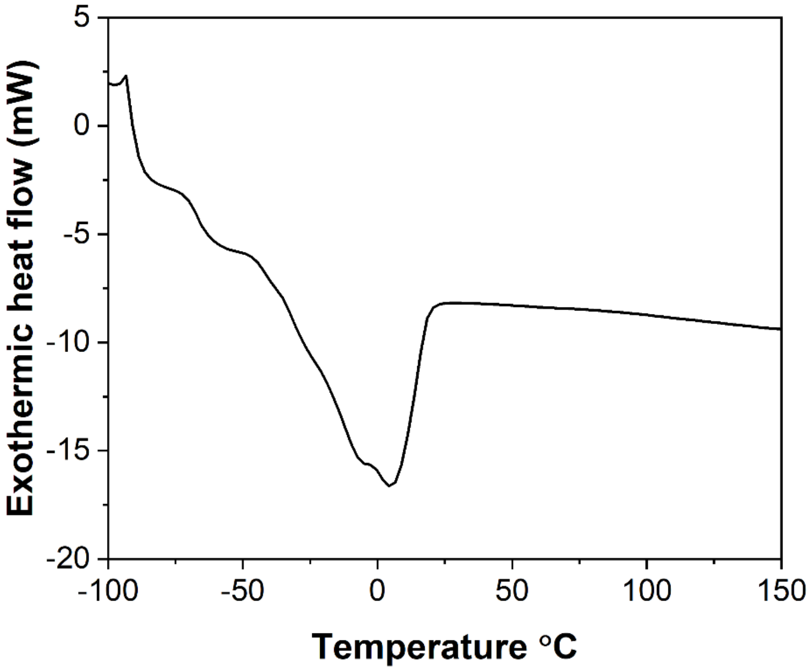

Figure S4. DSC trace of PEGDGE.

TGA analysis

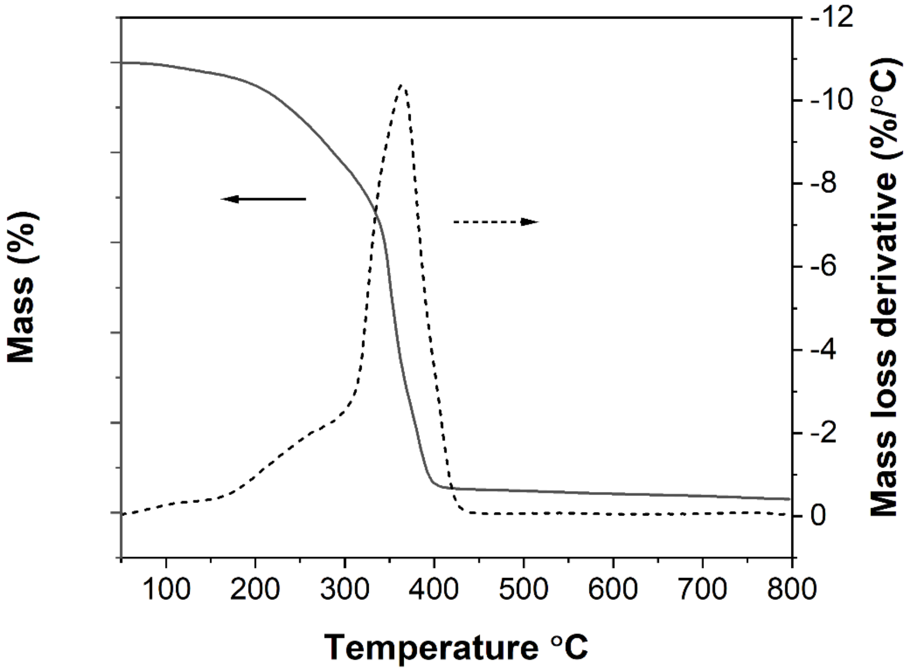

Figure S5. TGA profile of PEGDGE.

Free swelling capacity

The ability of the lignin-based crosslinked membranes to retain water upon soaking was evaluated in terms of their free swelling capacity (FSC). In particular, membranes obtained at different OL/PEGDGE weight ratios were immersed in water at 25 °C for 120 min and their increase in mass was assessed. As observed in **Figure S6**, lignin-based membranes incorporating a higher PEGDGE content (i.e., LM_0.5 and LM_0.7) were shown to exhibit the fastest swelling rate, in addition to reaching the highest equilibrium FSC value of around 2.5 $g_{H2O}/g_{membrane}$ after 2 min. Conversely, a significantly slower swelling rate was observed in LM_1 and LM_2, together with a markedly lower maximum FSC value, found in both cases to be slightly below 1.5 $g_{H2O}/g_{membrane}$ after 120 min of immersion (it is worth mentioning that 1.5 g of electrolyte is sufficient to assure a complete wetting of both the photoanode and the membrane in DSSC, as will be discussed in the following sections).[S10] These different behaviors may be attributed to the twofold effect of the ethoxy-based structure of PEGDGE on the swelling response of these lignin-based membranes, which provides an increasingly more marked hydrophilic character to the material and enables a higher macromolecular network mobility, as previously discussed based on DSC analyses. However, such combined effect on FSC was no longer observed at OL/PEGDGE mass ratios below 0.7, which appears to be a threshold value for water absorption in these systems. It is worth noting that the ultimate FSC values determined for all the lignin-based membranes lie within the typical ranges of common polymeric membranes used for traditional DSSC electrolytes.[S11,S12]

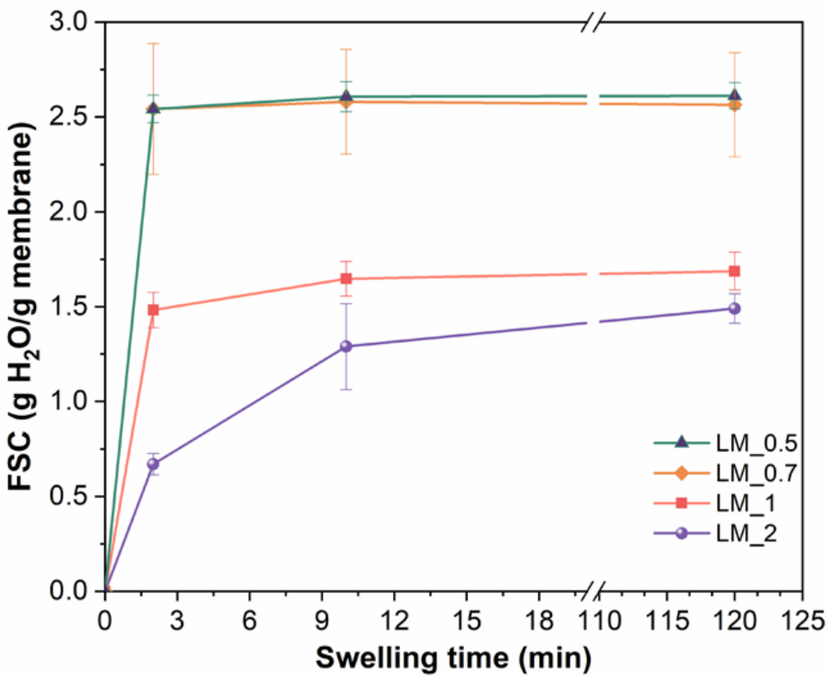

Figure S6. Free swelling capacity curves in water at 25 °C.

Incident photon-to-current efficiency (IPCE)

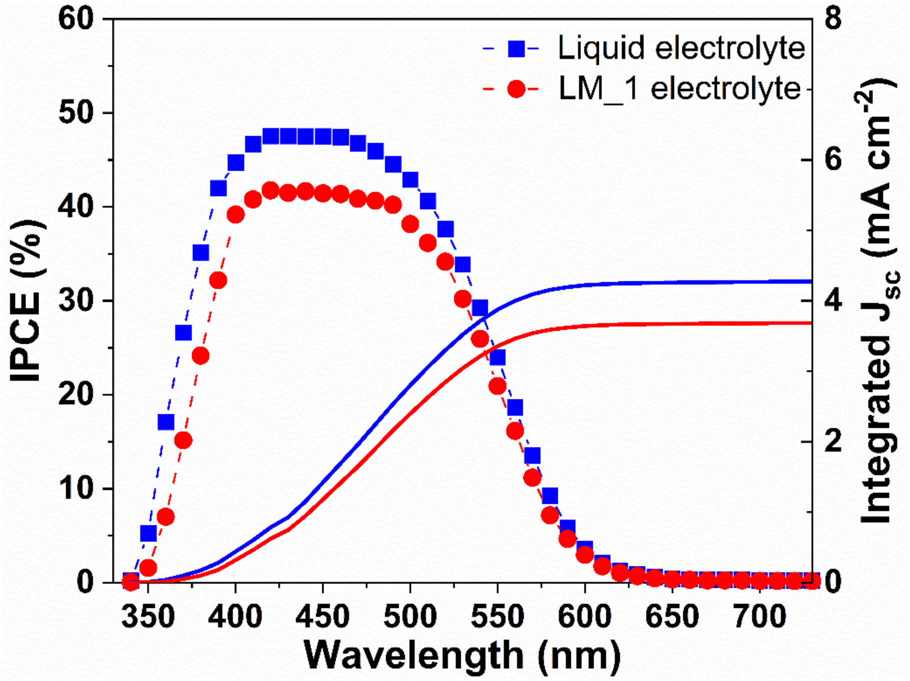

Figure S7. Incident photon-to-current efficiency (IPCE) measurements for LM_1-based DSSC devices, in comparison with benchmark liquid-state DSSC devices.

Swelling tests in liquid electrolyte

Swelling tests were performed on the different lignin-based membranes by dipping the pre-weighted dry materials in the aqueous electrolyte (see *Experimental section* for details on composition) at 25 ºC for 7 days (time required to achieve swelling equilibrium). After this time period, the soaked membrane was rinsed off gently and weighted.

The volume fraction of polymer in the swollen membrane ($\tilde{v}$) was determined gravimetrically following Equation S2:

$$\tilde{v} = \frac{m_d/\rho_d}{m_d/\rho_d + (m_w - m_d)/\rho_s} \tag{S2}$$

where ρ_s is the density of the swelling liquid (electrolyte), ρ_d the density of the dry membrane, m_w the mass of the swollen wet membrane and m_d its dry mass.

The value of ρ_d was estimated at each lignin/PEGDGE wt./wt. ratio considering the following equation:

$$\rho_d = w_{LIG} \cdot \rho_{LIG} + w_{PEGDGE} \cdot \rho_{PEGDGE} \tag{S3}$$

where w_{LIG} and w_{PEGDGE} are the mass fraction of lignin and PEGDGE in the membrane, respectively, while ρ_{LIG} and ρ_{PEGDGE} are the density of lignin (0.95 g/cm^3) and the density of PEGDGE (1.14 g/cm^3).

The volumetric free-swelling capacity (FSC_{vol}) was obtained after the swelling tests according to Equation S4:

$$FSC_{vol} = \frac{(m_w - m_d)/\rho_s}{m_d/\rho_d} \tag{S4}$$

Table S1 summarizes the main characteristic values obtained from swelling tests for the different lignin/PEGDGE membrane formulations.

Table S1. Volume fraction of the solid membrane in the swollen material ($\tilde{v}$), volume fraction of PEGDGE in the membrane (φ_{PEGDGE}) and volumetric free-swelling capacity (FSC_{vol}) of the membrane, all at varying lignin/PEGDGE wt./wt. ratio.

	$\tilde{v}$ [-]	FSC_{vol} [-]	φ_{PEGDGE} [-]
**LM_0.5**	0.27	2.69	0.63
**LM_0.7**	0.28	2.55	0.54
**LM_1**	0.37	1.67	0.45
**LM_2**	0.40	1.52	0.29

Crosslinking reaction

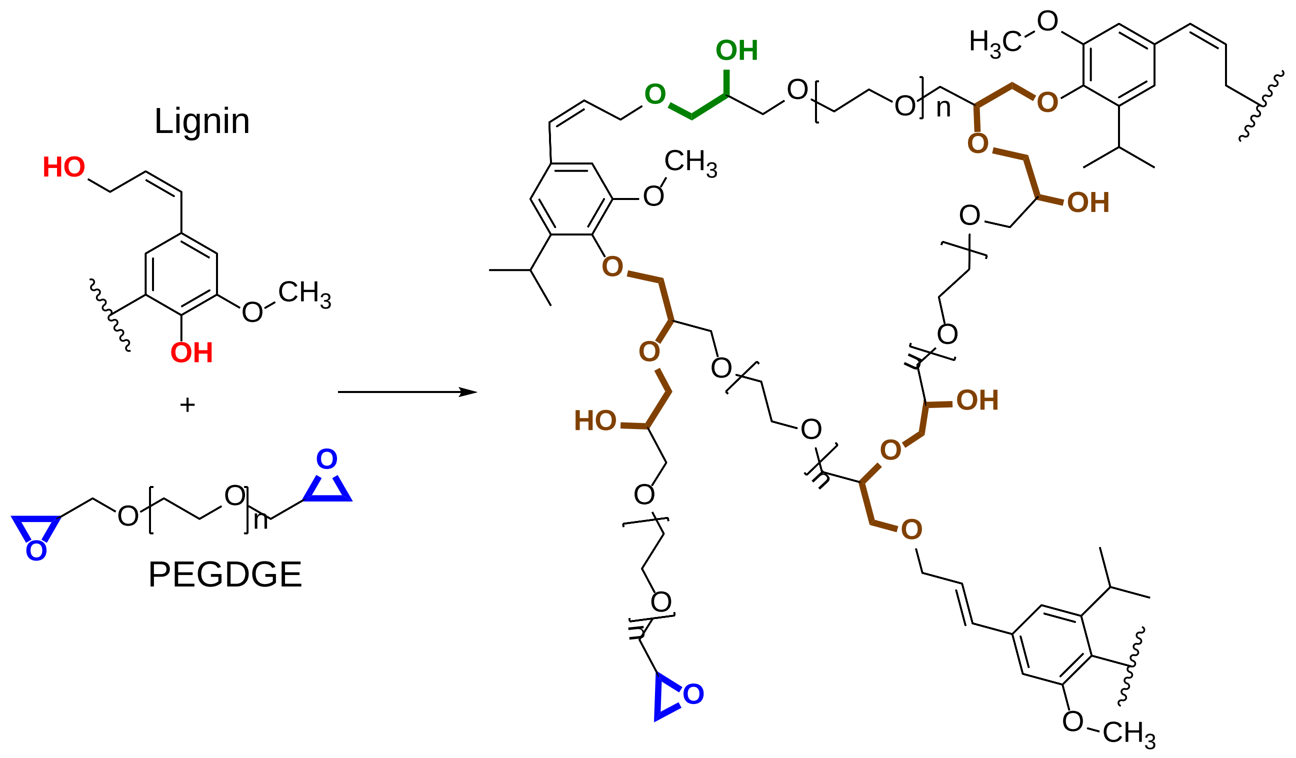

Figure S8. Reaction between PEGDGE and secondary hydroxyl groups resulting from the epoxy-ring opening reaction.

Additional electrochemical characterization

To further investigate the impact of electrolyte mass transport kinetics and macromolecular arrangement on device performance, transient photocurrent measurements under different light intensities were performed on DSSCs incorporating membranes with extreme compositions (LM_0.5 and LM_2, with highest and lowest amount of PEGDGE, respectively). As shown in **Figure S9**a, the last two photocurrent signals recorded at 0.8 and 1.0 sun highlight a clear mass transport limitation for both devices. This indicates that these electrolyte systems would perform better under medium and low irradiation intensity levels (i.e., below 0.6 sun), in line with the most preferable application scenarios currently proposed for commercial DSSCs that include integration in windows, indoor-environment powering and portable electronics.[S13,S14] Interestingly, the transient photocurrent measurements under different light intensities revealed two different device responses for LM_0.5 and LM_2 systems. In particular, the signal associated to the device

incorporating the high-PEGDGE-content membrane (LM_0.5) appears to anticipate its photocurrent decay with respect to LM_2 under all illumination conditions. This behavior may be correlated to the more entangled structure likely present in LM_0.5 as a result of the increased concentration of PEGDGE, which is expected to hinder more effectively the diffusion of the redox couple between the electrodes. The same devices were also tested using open-circuit voltage decay (OCVD) measurements to investigate the effect of membrane formulation on recombination rate at the photoanode/electrolyte interface. To this end, solar cells were kept under constant irradiation and open-circuit conditions, then the illumination was switched off and the voltage decay was recorded as a function of time. Photogenerated electrons can undergo recombination under these conditions, thus lowering their population to a dark equilibrium state. In the resulting curves (**Figure S9**b), no evident differences were observed between samples incorporating membranes with high (LM_0.5) and low (LM_2) PEGDGE content, indicating a negligible influence of the chemical composition of the QS electrolyte on the recombination phenomena occurring at the electrolyte/photoanode interface. Indeed, these results are in close agreement with the V_{oc} values plotted in **Figure 6**b in the main text, which were found to remain rather stable irrespective of the OL/PEGDGE relative proportions.

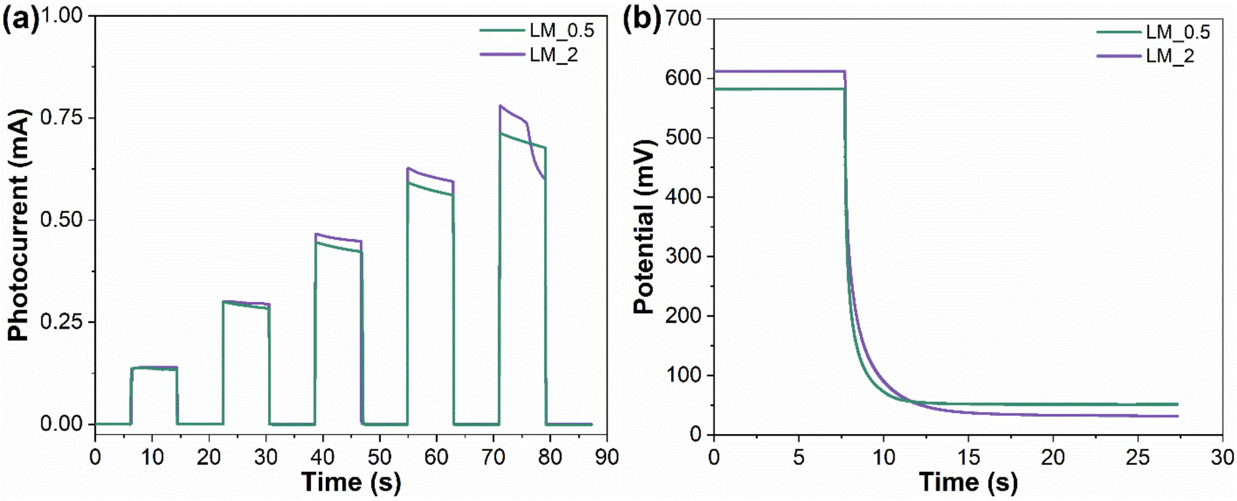

Figure S9. a) Transient photocurrent measurements at different light intensities (from left to right, irradiation intensity increases from 0.2 to 1.0 sun) for two DSSCs assembled with different lignin-based QS electrolytes. b) OCVD curves for the same devices.

Electrochemical impedance spectroscopy (EIS) data

Table S2. List of parameters obtained from the interpolation of the experimental EIS spectra of the most efficient devices.

	**LM_2**	**LM_1**	**LM_0.7**
R_s	18.7 ± 0.1	17.7 ± 0.2	19.4 ± 0.2
R_{CE}	23.1 ± 1.1	22.8 ± 0.9	27.4 ± 1.7
$C_{CE} \times 10^{-5}$	8.5 ± 0.3	7.8 ± 0.6	7.0 ± 1.0
R_d	41.4 ± 1.1	27.4 ± 0.8	20.7 ± 0.8
R_t	1.56 ± 0.18	1.51 ± 0.08	1.37 ± 0.12
R_{rec}	45.7 ± 6.2	37.5 ± 5.3	41.8 ± 6.3
$C_{\square} \times 10^{-4}$	1.74 ± 0.38	2.59 ± 0.51	3.80 ± 0.21

UV-VIS absorption

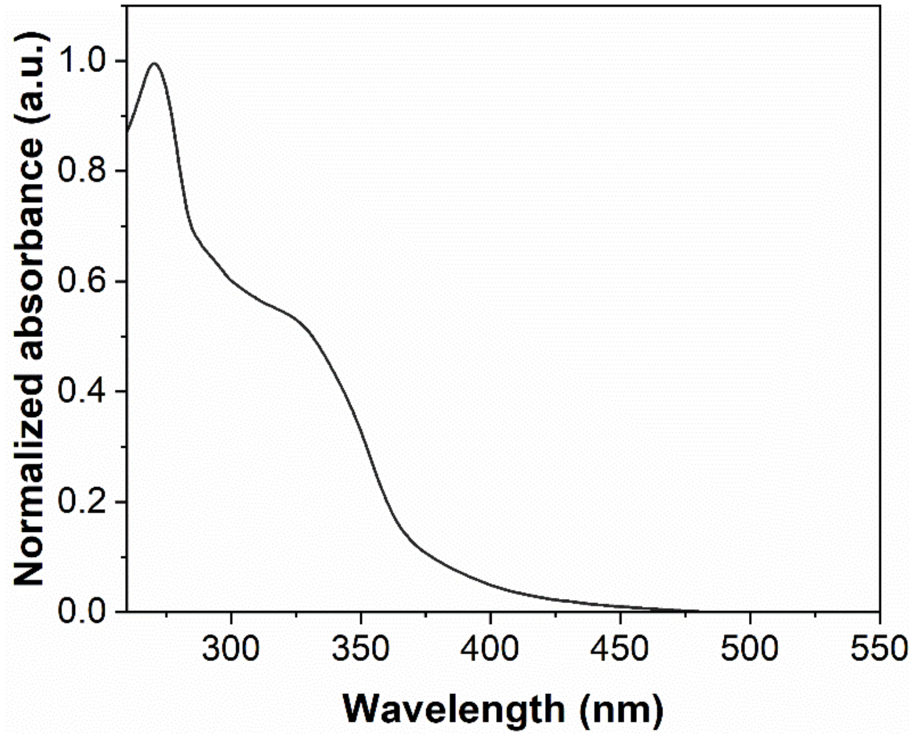

Figure S10. UV-vis spectrum of the lignin used in this work.

References

(S1) Balakshin, M.; Capanema, E. On the Quantification of Lignin Hydroxyl Groups With 31P and 13C NMR Spectroscopy. *J. Wood Chem. Technol.* **2015**, *35* (3), 220–237. https://doi.org/10.1080/02773813.2014.928328.

(S2) Bonomo, M.; Congiu, M.; De Marco, M. L.; Dowling, D. P.; Di Carlo, A.; Graeff, C. F. O.; Dini, D. Limits on the Use of Cobalt Sulfide as Anode of P-Type Dye-Sensitized Solar Cells. *J. Phys. D. Appl. Phys.* **2017**, *50* (21), 215501. https://doi.org/10.1088/1361-6463/aa6a79.

(S3) Glasser, W. G. About Making Lignin Great Again—Some Lessons From the Past. *Front. Chem.* **2019**, *7*, 565. https://doi.org/10.3389/fchem.2019.00565.

(S4) Griffini, G.; Passoni, V.; Suriano, R.; Levi, M.; Turri, S. Polyurethane Coatings Based on Chemically Unmodified Fractionated Lignin. *ACS Sustain. Chem. Eng.* **2015**, *3* (6), 1145–1154. https://doi.org/10.1021/acssuschemeng.5b00073.

(S5) Babuponnusami, A.; Muthukumar, K. A Review on Fenton and Improvements to the Fenton Process for Wastewater Treatment. *J. Environ. Chem. Eng.* **2014**, *2* (1), 557–572. https://doi.org/10.1016/j.jece.2013.10.011.

(S6) Pignatello, J. J.; Oliveros, E.; MacKay, A. Advanced Oxidation Processes for Organic Contaminant Destruction Based on the Fenton Reaction and Related Chemistry. *Crit. Rev. Environ. Sci. Technol.* **2006**, *36* (1), 1–84. https://doi.org/10.1080/10643380500326564.

(S7) Passauer, L.; Fischer, K.; Liebner, F. Preparation and Physical Characterization of Strongly Swellable Oligo(Oxyethylene) Lignin Hydrogels. *Holzforschung* **2011**, *65* (3), 309–317. https://doi.org/https://doi.org/10.1515/hf.2011.044.

(S8) Ghorbani, M.; Konnerth, J.; Budjav, E.; Requejo, A.; Zinovyev, G.; Van Herwijnen, H.; Edler, M.; Griesser, T.; Liebner, F. Ammoxidized Fenton-Activated Pine Kraft Lignin Accelerates Synthesis and Curing of Resole Resins. *Polymers (Basel).* **2017**, *9*, 43. https://doi.org/10.3390/polym9020043.

(S9) Passauer, L.; Fischer, K.; Liebner, F. Activation of Pine Kraft Lignin by Fenton-Type Oxidation for

Cross-Linking with Oligo(Oxyethylene) Diglycidyl Ether. *Holzforschung* **2011**, *65* (3), 319–326. https://doi.org/https://doi.org/10.1515/hf.2011.045.

(S10) Bella, F.; Galliano, S.; Falco, M.; Viscardi, G.; Barolo, C.; Grätzel, M.; Gerbaldi, C. Approaching Truly Sustainable Solar Cells by the Use of Water and Cellulose Derivatives. *Green Chem.* **2017**, *19* (4), 1043–1051. https://doi.org/10.1039/C6GC02625G.

(S11) Pang, H.-W.; Yu, H.-F.; Huang, Y.-J.; Li, C.-T.; Ho, K.-C. Electrospun Membranes of Imidazole-Grafted PVDF-HFP Polymeric Ionic Liquids for Highly Efficient Quasi-Solid-State Dye-Sensitized Solar Cells. *J. Mater. Chem. A* **2018**, *6* (29), 14215–14223. https://doi.org/10.1039/C8TA01215F.

(S12) Shanti, R.; Bella, F.; Salim, Y. S.; Chee, S. Y.; Ramesh, S.; Ramesh, K. Poly(Methyl Methacrylate-Co-Butyl Acrylate-Co-Acrylic Acid): Physico-Chemical Characterization and Targeted Dye Sensitized Solar Cell Application. *Mater. Des.* **2016**, *108*, 560–569. https://doi.org/https://doi.org/10.1016/j.matdes.2016.07.021.

(S13) Zhang, K.; Qin, C.; Yang, X.; Islam, A.; Zhang, S.; Chen, H.; Han, L. High-Performance, Transparent, Dye-Sensitized Solar Cells for See-Through Photovoltaic Windows. *Adv. Energy Mater.* **2014**, *4* (11), 1301966. https://doi.org/10.1002/aenm.201301966.

(S14) Venkatesan, S.; Lin, W.-H.; Teng, H.; Lee, Y.-L. High-Efficiency Bifacial Dye-Sensitized Solar Cells for Application under Indoor Light Conditions. *ACS Appl. Mater. Interfaces* **2019**, *11* (45), 42780–42789. https://doi.org/10.1021/acsami.9b14876.
